# (*E*)-1-Ferrocenyl-3-phenyl­prop-2-en-1-one

**DOI:** 10.1107/S1600536808020059

**Published:** 2008-07-05

**Authors:** Yong-Hong Liu, Jian-Feng Liu, Pan-Ming Jian, Xiao-Lan Liu

**Affiliations:** a College of Chemistry and Chemical Engineering, Yangzhou University, Yangzhou 225002, People’s Republic of China; b Technology Center, Jiuquan Iron and Steel (Group) Co. Ltd, Jiayuguan 735100, People’s Republic of China

## Abstract

The title compound, [Fe(C_5_H_5_)(C_14_H_11_O)], exists as the *E* isomer, and the substituent is fully conjugated with the attached five-membered ring. In the ferrocene unit, the substituted cyclo­penta­dienyl ring (Cps) plane and unsubstituted cyclo­penta­dienyl ring (Cp) plane are almost parallel, and the C atoms in Cp and Cps are in an eclipsed conformation. In the crystal structure, mol­ecules are linked into *C*(5) chains *via* inter­molecular C—H⋯O hydrogen bonds, and neighbouring chains are assembled into sheets by inter­molecular C—H⋯π(arene) hydrogen bonds along the *c* axis.

## Related literature

For related literature, see: Bernstein *et al.* (1995 ▶); Edwards *et al.* (1975 ▶); Huang *et al.* (1998 ▶); Liang *et al.* (1998 ▶); Liu *et al.* (2001 ▶, 2003 ▶, 2008 ▶); Shi *et al.* (2004 ▶); Yarishkin *et al.* (2008 ▶); Zhai *et al.* (1999 ▶).
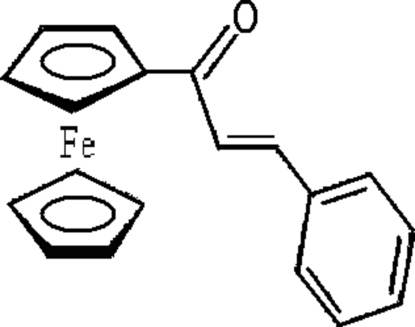


## Experimental

### 

#### Crystal data

[Fe(C_5_H_5_)(C_14_H_11_O)]
*M* *_r_* = 316.17
Orthorhombic, 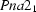

*a* = 22.717 (3) Å
*b* = 5.8173 (9) Å
*c* = 11.1789 (17) Å
*V* = 1477.3 (4) Å^3^
*Z* = 4
Mo *K*α radiation
μ = 1.02 mm^−1^
*T* = 296 (2) K
0.32 × 0.28 × 0.27 mm

#### Data collection

Bruker SMART 1000 CCD diffractometer
Absorption correction: multi-scan (*SADABS*; Bruker, 2007 ▶) *T*_min_ = 0.272, *T*_max_ = 0.318 (expected range = 0.650–0.760)
9864 measured reflections
2547 independent reflections
2409 reflections with *I* > 2σ(*I*)
*R*_int_ = 0.024

#### Refinement

*R*[*F* ^2^ > 2σ(*F* ^2^)] = 0.022
*wR*(*F* ^2^) = 0.055
*S* = 1.00
2547 reflections
190 parameters
1 restraint
H-atom parameters constrained
Δρ_max_ = 0.11 e Å^−3^
Δρ_min_ = −0.31 e Å^−3^
Absolute structure: Flack (1983 ▶), 1181 Friedel pairs
Flack parameter: 0.013 (16)

### 

Data collection: *SMART* (Bruker, 2007 ▶); cell refinement: *SAINT* (Bruker, 2007 ▶); data reduction: *SAINT*; program(s) used to solve structure: *SHELXS97* (Sheldrick, 2008 ▶); program(s) used to refine structure: *SHELXL97* (Sheldrick, 2008 ▶); molecular graphics: *PLATON* (Spek, 2003 ▶); software used to prepare material for publication: *SHELXL97*.

## Supplementary Material

Crystal structure: contains datablocks I, global. DOI: 10.1107/S1600536808020059/om2243sup1.cif

Structure factors: contains datablocks I. DOI: 10.1107/S1600536808020059/om2243Isup2.hkl

Additional supplementary materials:  crystallographic information; 3D view; checkCIF report

## Figures and Tables

**Table 1:** Dihedral angles (°) for selected planes

	Atoms defining plane	1-Plane	2-Plane	Cp plane
1-Plane	C11–C13/O1	–	–	–
2-Plane	C14–C19	33.0 (1)	–	–
Cp plane	C1–C5	17.9 (2)	50.6 (4)	–
Cps plane	C6–C10	17.0 (1)	49.9 (1)	1.8 (1)

**Table 2:** Hydrogen-bond geometry (Å, °) δ is the angle that the C1/H1 group makes with the normal to the Cp plane, and *Cg*3 is the centroid of the Cp ring.

D—H⋯A	D—H	H⋯A	D⋯A	D—H⋯A
C9—H9⋯O1^i^	0.98	2.67	3.538 (3)	148
C1—H1⋯*Cg*3^ii^	0.98	2.75	3.596 (2)	145 (δ = 64)

Symmetry codes: (i) 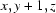; (ii) 
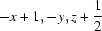
.

**Fig. 1.**
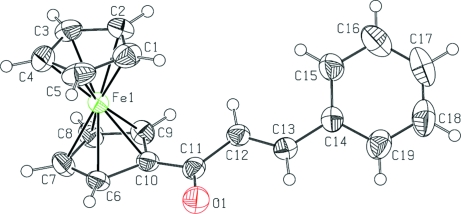
The molecular structure of the title compound, showing 50% probability ellipsoids.

**Fig. 2.**
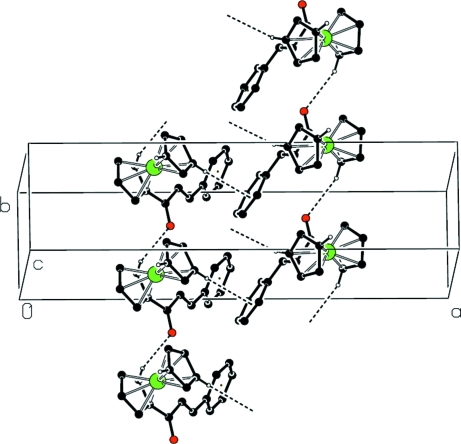
Part of the crystal structure of the title compound, showing the inter-molecular hydrogen bonds of C–H···O and C–H···π as dashed lines. For the sake of clarity, H atoms not involved in hydrogen bonding have been omitted.

## supplementary crystallographic information

### Comment

Chalcone and its derivatives, as a natural products, have shown stronge antibacterial, antifungal, antitumor and anti-inflammatory properties, especially antileishmanial, and antimalarial (Zhai *et al.*, 1999; Liu *et al.*, 2001, 2003). Some chalcones demonstrated the ability to block voltage-dependent potassium channels (Yarishkin *et al.*, 2008). It was proved that the replacement of the aromatic group by the ferrocenyl moiety in penicillins and cephalosporins could improve their antibiotic activity (Edwards *et al.*, 1975). As ongoing research (Liu *et al.*, 2008; Shi *et al.*, 2004; Liang *et al.*, 1998), we report herein the structure of the title compound.

The molecule of the title compound exists as the most stable configuration of (*E*)-isomer (Fig.1), and all carbon atoms are *sp*^2^-hybridized. Although the carbon atoms and a oxygen atom form a large conjugated system, the Cps (the substituted cyclopentadienyl ring) plane and 1-plane (defined by the atoms of C11, C12, O1 and C13) and 2-plane (the phenyl ring plane) are not coplanar (Table 1). In the ferrocene moiety, the Cps plane and Cp (the unsubstituted cyclopentadienyl ring) plane are almost parallel, and the carbon atoms of Cp and Cps are in the eclipsed conformation. The Fe atom is slightly nearer to the Cps plane because the Fe–Cgs and Fe–*Cg* distances are 1.651 (1) and 1.658 (1) Å, respectively, where Cgs and *Cg* are the centroids of Cps and Cp, respectively. The Cgs-Fe-Cg angle is 178.0 (2)°.

In its packing structure, the molecules are linked into *C(5)* (Bernstein *et al.*, 1995) chains *via *C–H···O inter-molecular hydrogen-bonds. Further more the chains and their neighboring inverse parallel chains are made up into sheets by C–H···π (arene) inter-molecular hydrogen-bonds along the *c* axis (Fig. 2, Table 2).

### Experimental

The title compound was synthesized according to the literature procedure (Huang *et al.* 1998). Crystals suitable for X-ray diffraction were obtained by slow evaporation of a solution of the solid in dichloromethane/ether (5:1 v/v) at room temperature over a period of 4 d.

### Refinement

After their location in a difference map, all H atoms were fixed geometrically at ideal positions and allowed to ride on the parent C atoms, with C—H distances of 0.93 – 0.98, and with *U_iso_*(H) values of *1.2U_eq_* (C).

### Crystal data

**Table:**

[Fe(C_5_H_5_)(C_14_H_11_O)]	*D*_x_ = 1.422 Mg m^−^^3^
*M**_r_* = 316.17	Melting point: 416 K
Orthorhombic, *P**n**a*2_1_	Mo *K*α radiation λ = 0.71073 Å
Hall symbol: P2c-2n	Cell parameters from 5183 reflections
*a* = 22.717 (3) Å	θ = 2.5–27.5º
*b* = 5.8173 (9) Å	µ = 1.02 mm^−^^1^
*c* = 11.1789 (17) Å	*T* = 296 (2) K
*V* = 1477.3 (4) Å^3^	Block, dark-red
*Z* = 4	0.32 × 0.28 × 0.27 mm
*F*_000_ = 656.0	

### Data collection

**Table:**

Bruker SMART 1000 CCD diffractometer	2547 independent reflections
Radiation source: fine-focus sealed tube	2409 reflections with *I* > 2σ(*I*)
Monochromator: graphite	*R*_int_ = 0.024
*T* = 296(2) K	θ_max_ = 25.0º
φ and ω scans	θ_min_ = 1.8º
Absorption correction: multi-scan(SADABS; Bruker, 2007)	*h* = −27→27
*T*_min_ = 0.272, *T*_max_ = 0.318	*k* = −6→6
9864 measured reflections	*l* = −13→13

### Refinement

**Table:**

Refinement on *F*^2^	Hydrogen site location: inferred from neighbouring sites
Least-squares matrix: full	H-atom parameters constrained
*R*[*F*^2^ > 2σ(*F*^2^)] = 0.022	*w* = 1/[σ^2^(*F*_o_^2^) + (0.0324*P*)^2^ + 0.1121*P*] where *P* = (*F*_o_^2^ + 2*F*_c_^2^)/3
*wR*(*F*^2^) = 0.055	(Δ/σ)_max_ = 0.001
*S* = 1.00	Δρ_max_ = 0.11 e Å^−^^3^
2547 reflections	Δρ_min_ = −0.31 e Å^−^^3^
190 parameters	Extinction correction: none
1 restraint	Absolute structure: Flack (1983), 1181 Friedel pairs
Primary atom site location: structure-invariant direct methods	Flack parameter: 0.013 (16)
Secondary atom site location: difference Fourier map	

### Special details

**Table:**

Geometry. All esds (except the esd in the dihedral angle between two l.s. planes) are estimated using the full covariance matrix. The cell esds are taken into account individually in the estimation of esds in distances, angles and torsion angles; correlations between esds in cell parameters are only used when they are defined by crystal symmetry. An approximate (isotropic) treatment of cell esds is used for estimating esds involving l.s. planes.
Refinement. Refinement of *F*^2^ against ALL reflections. The weighted *R*-factor *wR* and goodness of fit *S* are based on *F*^2^, conventional *R*-factors *R* are based on *F*, with *F* set to zero for negative *F*^2^. The threshold expression of *F*^2^ > σ(*F*^2^) is used only for calculating *R*-factors(gt) *etc*. and is not relevant to the choice of reflections for refinement. *R*-factors based on *F*^2^ are statistically about twice as large as those based on *F*, and *R*- factors based on ALL data will be even larger.

### Fractional atomic coordinates and isotropic or equivalent isotropic displacement parameters (Å^2^)

**Table:**

	*x*	*y*	*z*	*U*_iso_*/*U*_eq_	
Fe1	0.306705 (9)	0.01465 (4)	0.47590 (5)	0.03375 (9)	
C1	0.39580 (9)	0.0389 (4)	0.4986 (2)	0.0520 (7)	
H1	0.4258	−0.0099	0.4410	0.062*	
C2	0.36997 (8)	0.2578 (4)	0.50508 (19)	0.0486 (5)	
H2	0.3789	0.3888	0.4530	0.058*	
C3	0.32944 (10)	0.2565 (4)	0.6003 (2)	0.0511 (5)	
H3	0.3050	0.3865	0.6257	0.061*	
C4	0.32990 (12)	0.0378 (5)	0.6524 (2)	0.0578 (7)	
H4	0.3059	−0.0117	0.7205	0.069*	
C5	0.37087 (11)	−0.0994 (4)	0.5899 (2)	0.0563 (6)	
H5	0.3806	−0.2602	0.6070	0.068*	
C6	0.26327 (8)	−0.2636 (4)	0.40577 (18)	0.0403 (4)	
H6	0.2711	−0.4253	0.4243	0.048*	
C7	0.22257 (8)	−0.1193 (4)	0.4653 (2)	0.0474 (5)	
H7	0.1978	−0.1633	0.5333	0.057*	
C8	0.22496 (9)	0.1022 (4)	0.4118 (2)	0.0464 (5)	
H8	0.2019	0.2363	0.4366	0.056*	
C9	0.26678 (8)	0.0985 (4)	0.31925 (18)	0.0396 (4)	
H9	0.2772	0.2278	0.2672	0.047*	
C10	0.29116 (8)	−0.1304 (3)	0.31345 (17)	0.0355 (4)	
C11	0.33810 (8)	−0.2129 (3)	0.23428 (18)	0.0379 (4)	
C12	0.37129 (9)	−0.0364 (3)	0.16579 (18)	0.0374 (4)	
H12	0.3723	0.1141	0.1938	0.045*	
C13	0.39948 (8)	−0.0888 (4)	0.06591 (18)	0.0381 (4)	
H13	0.3976	−0.2411	0.0409	0.046*	
C14	0.43347 (7)	0.0699 (4)	−0.0094 (2)	0.0390 (4)	
C15	0.45591 (9)	0.2760 (4)	0.0339 (2)	0.0527 (6)	
H15	0.4490	0.3180	0.1129	0.063*	
C16	0.48835 (10)	0.4192 (4)	−0.0396 (3)	0.0685 (8)	
H16	0.5037	0.5557	−0.0096	0.082*	
C17	0.49790 (11)	0.3598 (6)	−0.1577 (3)	0.0731 (9)	
H17	0.5196	0.4565	−0.2071	0.088*	
C18	0.47553 (11)	0.1595 (6)	−0.2020 (2)	0.0706 (8)	
H18	0.4815	0.1212	−0.2819	0.085*	
C19	0.44402 (11)	0.0134 (4)	−0.1287 (2)	0.0551 (7)	
H19	0.4297	−0.1243	−0.1592	0.066*	
O1	0.34891 (7)	−0.4180 (3)	0.22424 (15)	0.0537 (4)	

### Atomic displacement parameters (Å^2^)

**Table:**

	*U*^11^	*U*^22^	*U*^33^	*U*^12^	*U*^13^	*U*^23^
Fe1	0.03735 (13)	0.03089 (14)	0.03302 (14)	−0.00503 (9)	0.00318 (15)	−0.00068 (16)
C1	0.0383 (9)	0.0610 (14)	0.0566 (19)	0.0006 (9)	−0.0049 (10)	−0.0147 (12)
C2	0.0470 (10)	0.0456 (12)	0.0533 (14)	−0.0170 (9)	−0.0010 (10)	−0.0027 (10)
C3	0.0575 (13)	0.0428 (13)	0.0530 (13)	−0.0073 (10)	0.0028 (11)	−0.0170 (11)
C4	0.0663 (15)	0.0747 (19)	0.0325 (12)	−0.0195 (13)	−0.0021 (11)	−0.0014 (12)
C5	0.0718 (14)	0.0403 (12)	0.0567 (15)	0.0009 (11)	−0.0294 (13)	−0.0010 (11)
C6	0.0472 (11)	0.0342 (11)	0.0394 (11)	−0.0132 (9)	−0.0020 (9)	0.0020 (9)
C7	0.0396 (9)	0.0548 (12)	0.0476 (12)	−0.0150 (8)	0.0054 (11)	0.0046 (14)
C8	0.0378 (11)	0.0476 (13)	0.0539 (12)	0.0006 (9)	0.0044 (9)	0.0009 (11)
C9	0.0393 (10)	0.0385 (11)	0.0408 (11)	0.0010 (9)	−0.0019 (8)	0.0050 (10)
C10	0.0413 (10)	0.0305 (10)	0.0346 (10)	−0.0065 (8)	−0.0032 (8)	0.0007 (8)
C11	0.0441 (10)	0.0383 (12)	0.0314 (10)	−0.0018 (8)	−0.0038 (8)	−0.0008 (9)
C12	0.0432 (10)	0.0308 (11)	0.0381 (12)	−0.0012 (8)	−0.0005 (8)	−0.0009 (8)
C13	0.0378 (9)	0.0379 (11)	0.0387 (11)	−0.0001 (9)	−0.0023 (8)	−0.0024 (9)
C14	0.0351 (8)	0.0464 (10)	0.0357 (12)	0.0030 (7)	0.0003 (9)	0.0035 (11)
C15	0.0501 (12)	0.0518 (14)	0.0563 (13)	−0.0021 (10)	0.0126 (10)	−0.0032 (11)
C16	0.0554 (12)	0.0530 (13)	0.097 (2)	−0.0041 (10)	0.0196 (16)	0.0093 (19)
C17	0.0561 (14)	0.087 (2)	0.076 (2)	0.0034 (15)	0.0202 (14)	0.0359 (17)
C18	0.0613 (14)	0.110 (2)	0.0409 (13)	0.0016 (16)	0.0054 (11)	0.0166 (15)
C19	0.0490 (13)	0.0759 (19)	0.0402 (14)	0.0005 (11)	−0.0025 (11)	−0.0008 (11)
O1	0.0756 (10)	0.0308 (8)	0.0548 (10)	0.0035 (7)	0.0113 (8)	−0.0010 (8)

### Geometric parameters (Å, °)

**Table:**

Fe1—C9	2.032 (2)	C7—H7	0.9800
Fe1—C10	2.033 (2)	C8—C9	1.405 (3)
Fe1—C2	2.0427 (19)	C8—H8	0.9800
Fe1—C3	2.044 (2)	C9—C10	1.443 (3)
Fe1—C1	2.045 (2)	C9—H9	0.9800
Fe1—C4	2.047 (2)	C10—C11	1.467 (3)
Fe1—C5	2.047 (2)	C11—O1	1.223 (2)
Fe1—C6	2.0513 (19)	C11—C12	1.486 (3)
Fe1—C8	2.055 (2)	C12—C13	1.323 (3)
Fe1—C7	2.0673 (17)	C12—H12	0.9300
C1—C2	1.404 (3)	C13—C14	1.469 (3)
C1—C5	1.417 (4)	C13—H13	0.9300
C1—H1	0.9800	C14—C15	1.390 (3)
C2—C3	1.407 (3)	C14—C19	1.393 (3)
C2—H2	0.9800	C15—C16	1.383 (4)
C3—C4	1.399 (3)	C15—H15	0.9300
C3—H3	0.9800	C16—C17	1.382 (4)
C4—C5	1.411 (4)	C16—H16	0.9300
C4—H4	0.9800	C17—C18	1.364 (4)
C5—H5	0.9800	C17—H17	0.9300
C6—C7	1.415 (3)	C18—C19	1.381 (4)
C6—C10	1.438 (3)	C18—H18	0.9300
C6—H6	0.9800	C19—H19	0.9300
C7—C8	1.421 (3)		
			
C9—Fe1—C10	41.60 (8)	C3—C4—H4	125.9
C9—Fe1—C2	106.59 (9)	C5—C4—H4	125.9
C10—Fe1—C2	123.51 (8)	Fe1—C4—H4	125.9
C9—Fe1—C3	122.27 (9)	C4—C5—C1	107.4 (2)
C10—Fe1—C3	159.53 (9)	C4—C5—Fe1	69.84 (14)
C2—Fe1—C3	40.28 (9)	C1—C5—Fe1	69.67 (12)
C9—Fe1—C1	122.18 (10)	C4—C5—H5	126.3
C10—Fe1—C1	108.15 (9)	C1—C5—H5	126.3
C2—Fe1—C1	40.18 (8)	Fe1—C5—H5	126.3
C3—Fe1—C1	67.54 (9)	C7—C6—C10	107.81 (18)
C9—Fe1—C4	158.46 (10)	C7—C6—Fe1	70.51 (11)
C10—Fe1—C4	158.91 (9)	C10—C6—Fe1	68.72 (10)
C2—Fe1—C4	67.63 (9)	C7—C6—H6	126.1
C3—Fe1—C4	40.01 (9)	C10—C6—H6	126.1
C1—Fe1—C4	67.71 (11)	Fe1—C6—H6	126.1
C9—Fe1—C5	158.82 (10)	C6—C7—C8	108.35 (19)
C10—Fe1—C5	123.03 (9)	C6—C7—Fe1	69.30 (10)
C2—Fe1—C5	67.91 (9)	C8—C7—Fe1	69.34 (10)
C3—Fe1—C5	67.65 (10)	C6—C7—H7	125.8
C1—Fe1—C5	40.52 (10)	C8—C7—H7	125.8
C4—Fe1—C5	40.34 (10)	Fe1—C7—H7	125.8
C9—Fe1—C6	69.22 (8)	C9—C8—C7	108.77 (19)
C10—Fe1—C6	41.22 (8)	C9—C8—Fe1	69.01 (11)
C2—Fe1—C6	161.06 (8)	C7—C8—Fe1	70.31 (11)
C3—Fe1—C6	157.58 (9)	C9—C8—H8	125.6
C1—Fe1—C6	125.31 (8)	C7—C8—H8	125.6
C4—Fe1—C6	122.96 (9)	Fe1—C8—H8	125.6
C5—Fe1—C6	108.95 (9)	C8—C9—C10	107.86 (18)
C9—Fe1—C8	40.22 (8)	C8—C9—Fe1	70.77 (12)
C10—Fe1—C8	68.56 (8)	C10—C9—Fe1	69.27 (11)
C2—Fe1—C8	121.33 (9)	C8—C9—H9	126.1
C3—Fe1—C8	107.15 (10)	C10—C9—H9	126.1
C1—Fe1—C8	157.07 (10)	Fe1—C9—H9	126.1
C4—Fe1—C8	123.54 (10)	C6—C10—C9	107.21 (17)
C5—Fe1—C8	160.26 (10)	C6—C10—C11	125.25 (18)
C6—Fe1—C8	68.13 (9)	C9—C10—C11	127.45 (18)
C9—Fe1—C7	68.19 (9)	C6—C10—Fe1	70.06 (11)
C10—Fe1—C7	68.41 (9)	C9—C10—Fe1	69.13 (11)
C2—Fe1—C7	157.00 (9)	C11—C10—Fe1	123.27 (13)
C3—Fe1—C7	122.13 (10)	O1—C11—C10	121.38 (18)
C1—Fe1—C7	161.47 (9)	O1—C11—C12	121.64 (18)
C4—Fe1—C7	108.53 (11)	C10—C11—C12	116.97 (17)
C5—Fe1—C7	124.88 (11)	C13—C12—C11	121.42 (19)
C6—Fe1—C7	40.19 (8)	C13—C12—H12	119.3
C8—Fe1—C7	40.35 (9)	C11—C12—H12	119.3
C2—C1—C5	108.1 (2)	C12—C13—C14	126.4 (2)
C2—C1—Fe1	69.83 (11)	C12—C13—H13	116.8
C5—C1—Fe1	69.81 (12)	C14—C13—H13	116.8
C2—C1—H1	125.9	C15—C14—C19	118.3 (2)
C5—C1—H1	125.9	C15—C14—C13	122.3 (2)
Fe1—C1—H1	125.9	C19—C14—C13	119.4 (2)
C1—C2—C3	107.9 (2)	C16—C15—C14	120.5 (3)
C1—C2—Fe1	69.98 (11)	C16—C15—H15	119.7
C3—C2—Fe1	69.92 (11)	C14—C15—H15	119.7
C1—C2—H2	126.1	C17—C16—C15	120.0 (3)
C3—C2—H2	126.1	C17—C16—H16	120.0
Fe1—C2—H2	126.1	C15—C16—H16	120.0
C4—C3—C2	108.4 (2)	C18—C17—C16	120.2 (2)
C4—C3—Fe1	70.09 (12)	C18—C17—H17	119.9
C2—C3—Fe1	69.80 (12)	C16—C17—H17	119.9
C4—C3—H3	125.8	C17—C18—C19	120.2 (2)
C2—C3—H3	125.8	C17—C18—H18	119.9
Fe1—C3—H3	125.8	C19—C18—H18	119.9
C3—C4—C5	108.2 (2)	C18—C19—C14	120.8 (2)
C3—C4—Fe1	69.90 (13)	C18—C19—H19	119.6
C5—C4—Fe1	69.82 (13)	C14—C19—H19	119.6
			
C9—Fe1—C1—C2	77.16 (16)	C10—C6—C7—C8	−0.3 (2)
C10—Fe1—C1—C2	120.82 (14)	Fe1—C6—C7—C8	58.49 (14)
C3—Fe1—C1—C2	−37.83 (14)	C10—C6—C7—Fe1	−58.80 (13)
C4—Fe1—C1—C2	−81.25 (15)	C9—Fe1—C7—C6	83.20 (13)
C5—Fe1—C1—C2	−119.2 (2)	C10—Fe1—C7—C6	38.26 (12)
C6—Fe1—C1—C2	163.27 (13)	C2—Fe1—C7—C6	164.2 (2)
C8—Fe1—C1—C2	43.1 (3)	C3—Fe1—C7—C6	−161.47 (13)
C7—Fe1—C1—C2	−163.1 (3)	C1—Fe1—C7—C6	−44.5 (4)
C9—Fe1—C1—C5	−163.61 (13)	C4—Fe1—C7—C6	−119.50 (15)
C10—Fe1—C1—C5	−119.96 (14)	C5—Fe1—C7—C6	−77.76 (17)
C2—Fe1—C1—C5	119.2 (2)	C8—Fe1—C7—C6	120.1 (2)
C3—Fe1—C1—C5	81.40 (15)	C9—Fe1—C7—C8	−36.94 (14)
C4—Fe1—C1—C5	37.98 (14)	C10—Fe1—C7—C8	−81.88 (15)
C6—Fe1—C1—C5	−77.51 (16)	C2—Fe1—C7—C8	44.1 (3)
C8—Fe1—C1—C5	162.3 (2)	C3—Fe1—C7—C8	78.39 (17)
C7—Fe1—C1—C5	−43.9 (4)	C1—Fe1—C7—C8	−164.6 (3)
C5—C1—C2—C3	0.4 (2)	C4—Fe1—C7—C8	120.36 (15)
Fe1—C1—C2—C3	59.90 (14)	C5—Fe1—C7—C8	162.10 (14)
C5—C1—C2—Fe1	−59.53 (14)	C6—Fe1—C7—C8	−120.1 (2)
C9—Fe1—C2—C1	−120.56 (15)	C6—C7—C8—C9	0.0 (2)
C10—Fe1—C2—C1	−78.18 (17)	Fe1—C7—C8—C9	58.43 (15)
C3—Fe1—C2—C1	118.8 (2)	C6—C7—C8—Fe1	−58.46 (14)
C4—Fe1—C2—C1	81.47 (16)	C10—Fe1—C8—C9	−38.75 (13)
C5—Fe1—C2—C1	37.73 (15)	C2—Fe1—C8—C9	78.34 (15)
C6—Fe1—C2—C1	−46.4 (3)	C3—Fe1—C8—C9	120.02 (14)
C8—Fe1—C2—C1	−161.85 (14)	C1—Fe1—C8—C9	47.3 (3)
C7—Fe1—C2—C1	166.3 (3)	C4—Fe1—C8—C9	160.81 (13)
C9—Fe1—C2—C3	120.67 (14)	C5—Fe1—C8—C9	−168.5 (2)
C10—Fe1—C2—C3	163.06 (13)	C6—Fe1—C8—C9	−83.26 (13)
C1—Fe1—C2—C3	−118.8 (2)	C7—Fe1—C8—C9	−120.2 (2)
C4—Fe1—C2—C3	−37.29 (14)	C9—Fe1—C8—C7	120.2 (2)
C5—Fe1—C2—C3	−81.03 (16)	C10—Fe1—C8—C7	81.47 (15)
C6—Fe1—C2—C3	−165.1 (3)	C2—Fe1—C8—C7	−161.44 (14)
C8—Fe1—C2—C3	79.38 (16)	C3—Fe1—C8—C7	−119.75 (15)
C7—Fe1—C2—C3	47.6 (3)	C1—Fe1—C8—C7	167.5 (2)
C1—C2—C3—C4	−0.2 (2)	C4—Fe1—C8—C7	−78.97 (17)
Fe1—C2—C3—C4	59.71 (15)	C5—Fe1—C8—C7	−48.3 (3)
C1—C2—C3—Fe1	−59.94 (14)	C6—Fe1—C8—C7	36.97 (14)
C9—Fe1—C3—C4	163.52 (14)	C7—C8—C9—C10	0.4 (2)
C10—Fe1—C3—C4	−163.4 (2)	Fe1—C8—C9—C10	59.58 (13)
C2—Fe1—C3—C4	−119.4 (2)	C7—C8—C9—Fe1	−59.23 (15)
C1—Fe1—C3—C4	−81.62 (17)	C10—Fe1—C9—C8	118.64 (17)
C5—Fe1—C3—C4	−37.62 (16)	C2—Fe1—C9—C8	−119.21 (13)
C6—Fe1—C3—C4	48.0 (3)	C3—Fe1—C9—C8	−78.08 (16)
C8—Fe1—C3—C4	122.12 (16)	C1—Fe1—C9—C8	−160.23 (13)
C7—Fe1—C3—C4	80.54 (17)	C4—Fe1—C9—C8	−48.3 (3)
C9—Fe1—C3—C2	−77.13 (15)	C5—Fe1—C9—C8	169.3 (2)
C10—Fe1—C3—C2	−44.0 (3)	C6—Fe1—C9—C8	80.31 (13)
C1—Fe1—C3—C2	37.74 (14)	C7—Fe1—C9—C8	37.05 (13)
C4—Fe1—C3—C2	119.4 (2)	C2—Fe1—C9—C10	122.15 (12)
C5—Fe1—C3—C2	81.74 (15)	C3—Fe1—C9—C10	163.28 (12)
C6—Fe1—C3—C2	167.4 (2)	C1—Fe1—C9—C10	81.12 (13)
C8—Fe1—C3—C2	−118.52 (14)	C4—Fe1—C9—C10	−166.9 (2)
C7—Fe1—C3—C2	−160.10 (14)	C5—Fe1—C9—C10	50.6 (3)
C2—C3—C4—C5	0.0 (3)	C6—Fe1—C9—C10	−38.33 (11)
Fe1—C3—C4—C5	59.53 (16)	C8—Fe1—C9—C10	−118.64 (17)
C2—C3—C4—Fe1	−59.53 (15)	C7—Fe1—C9—C10	−81.59 (12)
C9—Fe1—C4—C3	−40.8 (3)	C7—C6—C10—C9	0.5 (2)
C10—Fe1—C4—C3	163.9 (2)	Fe1—C6—C10—C9	−59.41 (13)
C2—Fe1—C4—C3	37.54 (14)	C7—C6—C10—C11	177.20 (18)
C1—Fe1—C4—C3	81.14 (15)	Fe1—C6—C10—C11	117.27 (19)
C5—Fe1—C4—C3	119.3 (2)	C7—C6—C10—Fe1	59.92 (14)
C6—Fe1—C4—C3	−160.25 (13)	C8—C9—C10—C6	−0.5 (2)
C8—Fe1—C4—C3	−76.15 (17)	Fe1—C9—C10—C6	60.00 (13)
C7—Fe1—C4—C3	−118.23 (15)	C8—C9—C10—C11	−177.12 (18)
C9—Fe1—C4—C5	−160.1 (2)	Fe1—C9—C10—C11	−116.59 (19)
C10—Fe1—C4—C5	44.6 (3)	C8—C9—C10—Fe1	−60.53 (14)
C2—Fe1—C4—C5	−81.75 (16)	C9—Fe1—C10—C6	−118.36 (16)
C3—Fe1—C4—C5	−119.3 (2)	C2—Fe1—C10—C6	164.95 (12)
C1—Fe1—C4—C5	−38.15 (14)	C3—Fe1—C10—C6	−162.5 (2)
C6—Fe1—C4—C5	80.46 (17)	C1—Fe1—C10—C6	123.29 (13)
C8—Fe1—C4—C5	164.56 (14)	C4—Fe1—C10—C6	48.3 (3)
C7—Fe1—C4—C5	122.48 (15)	C5—Fe1—C10—C6	81.11 (15)
C3—C4—C5—C1	0.2 (3)	C8—Fe1—C10—C6	−80.85 (12)
Fe1—C4—C5—C1	59.80 (15)	C7—Fe1—C10—C6	−37.34 (12)
C3—C4—C5—Fe1	−59.57 (16)	C2—Fe1—C10—C9	−76.69 (14)
C2—C1—C5—C4	−0.4 (2)	C3—Fe1—C10—C9	−44.1 (3)
Fe1—C1—C5—C4	−59.91 (16)	C1—Fe1—C10—C9	−118.35 (12)
C2—C1—C5—Fe1	59.54 (15)	C4—Fe1—C10—C9	166.7 (2)
C9—Fe1—C5—C4	159.8 (2)	C5—Fe1—C10—C9	−160.53 (13)
C10—Fe1—C5—C4	−162.47 (14)	C6—Fe1—C10—C9	118.36 (16)
C2—Fe1—C5—C4	80.99 (15)	C8—Fe1—C10—C9	37.51 (12)
C3—Fe1—C5—C4	37.32 (14)	C7—Fe1—C10—C9	81.02 (12)
C1—Fe1—C5—C4	118.4 (2)	C9—Fe1—C10—C11	121.9 (2)
C6—Fe1—C5—C4	−118.98 (15)	C2—Fe1—C10—C11	45.2 (2)
C8—Fe1—C5—C4	−41.1 (3)	C3—Fe1—C10—C11	77.8 (3)
C7—Fe1—C5—C4	−77.16 (18)	C1—Fe1—C10—C11	3.54 (19)
C9—Fe1—C5—C1	41.4 (3)	C4—Fe1—C10—C11	−71.5 (3)
C10—Fe1—C5—C1	79.12 (14)	C5—Fe1—C10—C11	−38.6 (2)
C2—Fe1—C5—C1	−37.42 (12)	C6—Fe1—C10—C11	−119.8 (2)
C3—Fe1—C5—C1	−81.09 (14)	C8—Fe1—C10—C11	159.39 (19)
C4—Fe1—C5—C1	−118.4 (2)	C7—Fe1—C10—C11	−157.09 (19)
C6—Fe1—C5—C1	122.61 (13)	C6—C10—C11—O1	15.5 (3)
C8—Fe1—C5—C1	−159.5 (3)	C9—C10—C11—O1	−168.5 (2)
C7—Fe1—C5—C1	164.43 (13)	Fe1—C10—C11—O1	103.5 (2)
C9—Fe1—C6—C7	−80.41 (14)	C6—C10—C11—C12	−165.28 (18)
C10—Fe1—C6—C7	−119.08 (18)	C9—C10—C11—C12	10.7 (3)
C2—Fe1—C6—C7	−160.9 (3)	Fe1—C10—C11—C12	−77.3 (2)
C3—Fe1—C6—C7	44.9 (3)	O1—C11—C12—C13	22.2 (3)
C1—Fe1—C6—C7	164.17 (16)	C10—C11—C12—C13	−157.06 (18)
C4—Fe1—C6—C7	79.60 (17)	C11—C12—C13—C14	179.80 (18)
C5—Fe1—C6—C7	122.05 (15)	C12—C13—C14—C15	21.4 (3)
C8—Fe1—C6—C7	−37.10 (14)	C12—C13—C14—C19	−158.8 (2)
C9—Fe1—C6—C10	38.67 (11)	C19—C14—C15—C16	−0.7 (3)
C2—Fe1—C6—C10	−41.8 (3)	C13—C14—C15—C16	179.1 (2)
C3—Fe1—C6—C10	164.0 (2)	C14—C15—C16—C17	1.0 (4)
C1—Fe1—C6—C10	−76.75 (15)	C15—C16—C17—C18	−0.2 (4)
C4—Fe1—C6—C10	−161.32 (13)	C16—C17—C18—C19	−1.0 (4)
C5—Fe1—C6—C10	−118.87 (13)	C17—C18—C19—C14	1.4 (4)
C8—Fe1—C6—C10	81.98 (12)	C15—C14—C19—C18	−0.5 (3)
C7—Fe1—C6—C10	119.08 (18)	C13—C14—C19—C18	179.6 (2)

### Table 1 Dihedral angles (°) for selected planes

**Table:**

	Atoms defining plane	1-Plane	2-Plane	Cp plane
1-Plane	C11–C13/O1			
2-Plane	C14–C19	33.0 (1)		
Cp plane	C1–C5	17.9 (2)	50.6 (4)	
Cps plane	C6–C10	17.0 (1)	49.9 (1)	1.8 (1)

### Table 2 Hydrogen-bond geometry (Å, °)

**Table:**

D—H···A	D—H	H···A	D···A	D—H···A
C9—H9···O1^i^	0.98	2.67	3.538 (3)	148
C1—H1···Cg3^ii^	0.98	2.75	3.596 (2)	145 (δ = 64)

δ is the angle the C1/H1 group makes with the normal to the Cp plane, and Cg3 is the centroid of the Cp ring. Symmetry codes: (i) x, y+1, z; (ii) -x+1, -y, z+1/2.
